# Supplementary material for: SOMAscan Proteomics Identifies Novel Plasma Proteins in Amyotrophic Lateral Sclerosis Patients
Source: Int J Mol Sci. 2023 Jan 18;24(3):1899. doi: 10.3390/ijms24031899 (PMC9916400; doi:10.3390/ijms24031899)
Supplement: Supplementary file 1 [file ijms-24-01899-s001.zip › ijms-2072241-supplementary.pdf]

Supplementary File

SOMAscan Proteomics Identifies Novel Plasma Proteins in Amyotrophic Lateral Sclerosis Patients

Elena Berrone [1,†], Giovanna Chiorino [2,†], Francesca Guana [2], Valerio Benedetti [1], Claudia Palmitessa [1], Marina Gallo [1], Andrea Calvo [3,4], Federico Casale [3], Umberto Manera [3,4], Alessandra Favole [1,*], Paola Crociara [1,5], Camilla Testori [1], Valerio Carta [1], Carlotta Tessarolo [1], Antonio D'Angelo [6], Giovanni De Marco [3,4], Maria Caramelli [1], Adriano Chiò [3,4,*], Cristina Casalone [1] and Cristiano Corona [1]

1 S.C. Neuroscienze, Istituto Zooprofilattico Sperimentale del Piemonte, Liguria e Valle d'Aosta, 10154 Turin, Italy
2 Cancer Genomics Laboratory, Fondazione Edo ed Elvo Tempia, 13900 Biella, Italy
3 Rita Levi Montalcini Department of Neuroscience, University of Turin, 10126 Turin, Italy
4 Neurology, Hospital Department of Neuroscience and Mental Health, Città della Salute e della Scienza Hospital of Turin, 10126 Turin, Italy
5 ASL TO4, 10034 Chivasso, Italy
6 Department of Veterinary Science, University of Turin, 10095 Grugliasco, Italy
* Correspondence: alessandra.favole@izsto.it (A.F.); adriano.chio@unito.it (A.C.)
† These authors contributed equally to this work.

Table S1. Upregulated and Downregulated proteins between ALS patients and neurologically healthy controls using SAM Analysis of SOMAscan Results.

**Increased in ALS patients**			
**Target Fullname**	**Entrez Gene Sym**	**logFC**	**p-value**
SH21A	SH2D1A	1.689103	0.019616
TIMP-3	TIMP3	1.292908	0.003370
PDGF-BB	PDGFB	1.205864	0.005048
TARC	CCL17	1.081839	0.003579
PKC-D	PRKCD	1.004368	0.011062
Gro-b/g	CXCL3 CXCL2	1.003084	0.009649
NID2	NID2	0.515259	0.011365
Nidogen	NID1	0.419618	0.012383
**Decreased in ALS patients**			
**Target Fullname**	**Entrez Gene Sym**	**logFC**	**p-value**
Ephrin-A2	EFNA2	-0.281950	0.024822
RGMB	RGMB	-0.313691	0.018977
VCAM-1	VCAM1	-0.315918	0.007538
sTie-1	TIE1	-0.334705	0.015354
TLR4	TLR4	-0.371138	0.006458
RGM-C	HJV	-0.388838	0.008869
IGF-I sR	IGF1R	-0.415958	0.025886
BMPR1A	BMPR1A	-0.449027	0.015265
LYPD3	LYPD3	-0.456034	0.011876
NCAM-L1	L1CAM	-0.482127	0.003169
BCAM	BCAM	-0.485299	0.031243
sTie-2	TEK	-0.488869	0.014479
Ephrin-A3	EFNA3	-0.514959	0.002038
MASP3	MASP1	-0.536970	0.011929
Kallikrein 7	KLK7	-0.553409	0.024028
Nectin-like protein 2	CADM1	-0.559252	0.045222
RET	RET	-0.566935	0.019690
IR	INSR	-0.594081	0.024830
TLR4:MD-2 complex	TLR4 LY96	-0.597382	0.007250
IL-13 Ra1	IL13RA1	-0.597611	0.014114
CD23	FCER2	-0.602595	0.046311
HAI-1	SPINT1	-0.620640	0.008536
sE-Selectin	SELE	-0.625872	0.024278
TECK	CCL25	-0.655796	0.041103
Carbonic anhydrase I	CA1	-0.703275	0.003678
ISLR2	ISLR2	-0.738526	0.000955
IL-5 Ra	IL5RA	-0.742331	0.026171
kallikrein 8	KLK8	-0.791363	0.021481
Growth hormone receptor	GHR	-0.820837	0.004480
BST1	BST1	-0.884313	0.037880
CYTT	CST2	-0.905443	0.017786
IL-18 BPa	IL18BP	-0.906512	0.001023
TF	F3	-1.041923	0.001738
ENPP7	ENPP7	-1.261712	0.003212

Table S2. Upregulated and Downregulated proteins between ALS patients and neurologically healthy controls using limma analysis of SOMAscan Results.

**Increased in ALS patients**			
**Target Fullname**	**Entrez Gene Sym**	**logFC**	**p-value**
Troponin T	TNNT2	1.477579	0.000924
TIMP-3	TIMP3	1.292908	0.003370
PDGF-BB	PDGFB	1.205864	0.005048
TARC	CCL17	1.081839	0.003579
Gro-b/g	CXCL3 CXCL2	1.003084	0.009649
DKK1	DKK1	0.826350	0.004899
CSRP3	CSRP3	0.786139	0.002050
Dkk-4	DKK4	0.685932	0.008682
**Decreased in ALS patients**			
**Target Fullname**	**Entrez Gene Sym**	**logFC**	**p-value**
TLR4:MD-2 complex	TLR4 LY96	-0.597382	0.007250
HAI-1	SPINT1	-0.620640	0.008536
Carbonic anhydrase I	CA1	-0.703275	0.003678
ISLR2	ISLR2	-0.738526	0.000955
Growth hormone receptor	GHR	-0.820837	0.004480
IL-1a	IL1A	-0.849595	0.003406
IL-18 BPa	IL18BP	-0.906512	0.001023
TF	F3	-1.041923	0.001738
ENPP7	ENPP7	-1.261712	0.003212

Table S3. Upregulated and Downregulated proteins between S-ALS patients and neurologically healthy controls using SAM Analysis of SOMAscan Results.

**Increased in ALS patients**			
**Target Fullname**	**Entrez Gene Sym**	**logFC**	**p-value**
TIMP-3	TIMP3	1.488049	0.003717
TARC	CCL17	1.451586	0.000576
Protein disulfide isomerase A3	PDIA3	1.020243	0.031587
AN32B	ANP32B	0.618600	0.002078
NID2	NID2	0.602565	0.011215
**Decreased in ALS patients**			
**Target Fullname**	**Entrez Gene Sym**	**logFC**	**p-value**
Ephrin-A2	EFNA2	-0.337748	0.021640
sTie-1	TIE1	-0.346598	0.031437
IL-16	IL16	-0.354345	0.054714
VCAM-1	VCAM1	-0.368661	0.007593
ULBP-2	ULBP2	-0.457169	0.010375
VEGF sR3	FLT4	-0.469542	0.004093
Ephrin-A3	EFNA3	-0.504992	0.008539
BMPR1A	BMPR1A	-0.514741	0.017360
BCAM	BCAM	-0.515190	0.050522
IGF-I	IGF1	-0.516672	0.013849
LYPD3	LYPD3	-0.532839	0.011828
Cathepsin S	CTSS	-0.546092	0.033920
sTie-2	TEK	-0.554973	0.017552
NCAM-L1	L1CAM	-0.571303	0.002630
IL-18 Ra	IL18R1	-0.602471	0.038568
TLR4:MD-2 complex	TLR4 LY96	-0.661724	0.010767
IL-13 Ra1	IL13RA1	-0.681839	0.016537
Kallikrein 7	KLK7	-0.696381	0.014740
granzyme A	GZMA	-0.710334	0.004400
RET	RET	-0.749046	0.007686
ISLR2	ISLR2	-0.787154	0.002363
IR	INSR	-0.791866	0.009677
IL-18 BPa	IL18BP	-0.844704	0.006945
sE-Selectin	SELE	-0.860835	0.007021
CD23	FCER2	-0.896461	0.009192
Myostatin	MSTN	-0.897505	0.056884
kallikrein 8	KLK8	-0.924181	0.021588
Lactoferrin	LTF	-0.957086	0.031620
Growth hormone receptor	GHR	-1.138476	0.000460

Table S4. Downregulated proteins between F-ALS patients and neurologically healthy controls using SAM Analysis of SOMAscan Results. No significantly up-regulated proteins were identified.

**Decreased in ALS patients**			
**Target Fullname**	**Entrez Gene Sym**	**logFC**	**p-value**
RNF43	RNF43	-0.403979	0.027766
TLR4	TLR4	-0.434531	0.006334
RGM-C	HFE2	-0.481616	0.006041
ISLR2	ISLR2	-0.689897	0.002233
Carbonic anhydrase I	CA1	-0.812518	0.002971
Discoidin domain receptor 2	DDR2	-1.138072	0.003942
TF	F3	-1.466182	0.000018
ENPP7	ENPP7	-1.498924	0.000856

Table S5. Upregulated and Downregulated proteins between S-ALS patients and neurologically healthy controls using limma Analysis of SOMAscan Results.

**Increased in ALS patients**			
**Target Fullname**	**Entrez Gene Sym**	**logFC**	**p-value**
TIMP-3	TIMP3	1.488049	0.003717
Troponin T	TNNT2	1.483767	0.003801
TARC	CCL17	1.451586	0.000576
G-CSF-R	CSF3R	1.193777	0.008583
IGFBP-2	IGFBP2	1.114518	0.003953
DKK1	DKK1	0.978497	0.004176
Dkk-4	DKK4	0.863833	0.004338
FGF-10	FGF10	0.754318	0.009419
LIMP II	SCARB2	0.628880	0.004092
AN32B	ANP32B	0.618600	0.002078
SDF-1	CXCL12	0.616372	0.003589
CD22	CD22	0.592353	0.009333
**Decreased in ALS patients**			
**Target Fullname**	**Entrez Gene Sym**	**logFC**	**p-value**
Nogo Receptor	RTN4R	-0.590989	0.001815
granzyme A	GZMA	-0.710334	0.004400
RET	RET	-0.749046	0.007686
ISLR2	ISLR2	-0.787154	0.002363
IR	INSR	-0.791866	0.009677
IL-18 BPa	IL18BP	-0.844704	0.006945
sE-Selectin	SELE	-0.860835	0.007021
CD23	FCER2	-0.896461	0.009192
Growth hormone receptor	GHR	-1.138476	0.000460

Table S6. Upregulated and Downregulated proteins between F-ALS patients and neurologically healthy controls using READAT Analysis of Somascan Results.

**Increased in ALS patients**			
**Target Fullname**	**Entrez Gene Sym**	**logFC**	**p-value**
PDE5A	PDE5A	2.515048	0.003031
M2-PK	PKM	2.282772	0.004186
AMPM2	METAP2	2.111687	0.008841
FER	FER	1.846274	0.008930
MAPK2	MAPKAPK2	1.693216	0.002242
CPNE1	CPNE1	1.667009	0.007142
DLRB1	DYNLRB1	1.655651	0.005362
Troponin T	TNNT2	1.471392	0.002702
PKC-D	PRKCD	1.426355	0.001771
EGF	EGF	1.385597	0.002806
Gro-b/g	CXCL3 CXCL2	1.213105	0.008527
C5a	C5	1.196144	0.008846
CAMK2A	CAMK2A	1.189491	0.003996
cGMP-stimulated PDE	PDE2A	1.012715	0.003776
C6	C6	0.941726	0.008187
CSRP3	CSRP3	0.845542	0.000344
Calcineurin B a	PPP3R1	0.807205	0.008612
**Decreased in ALS patients**			
**Target Fullname**	**Entrez Gene Sym**	**logFC**	**p-value**
calreticulin	CALR	-0.583783	0.004835
ISLR2	ISLR2	-0.689897	0.002233
TECK	CCL25	-0.808331	0.009408
Carbonic anhydrase I	CA1	-0.812518	0.002971
BMP-6	BMP6	-0.885880	0.000407
IL-18 BPa	IL18BP	-0.968319	0.001700
Discoidin domain receptor 2	DDR2	-1.138072	0.003942
a2-Macroglobulin	A2M	-1.438991	0.004912
TF	F3	-1.466182	0.000018
ENPP7	ENPP7	-1.498924	0.000856
Thrombin	F2	-1.509842	0.007796
C3b	C3	-1.703522	0.003412

Table S7. List of GO Annotation for TARC, TIMP-3, NID1 and NID2. Abbreviations: P-Pathway, F-Function; C- Cellular.

TARC

**Qualifier**	**GO Term**	**GO Name**	**Reference**	**GO Aspect**
involved_in	GO:0043547	positive regulation of GTPase activity	PMID:21873635	P
enables	GO:0008009	chemokine activity	PMID:21873635	F
involved_in	GO:0048247	lymphocyte chemotaxis	PMID:21873635	P
is_active_in	GO:0005615	extracellular space	PMID:21873635	C

involved_in	GO:0070098	chemokine-mediated signaling pathway	PMID:21873635	P
involved_in	GO:0070374	positive regulation of ERK1 and ERK2 cascade	PMID:21873635	P
involved_in	GO:0071346	cellular response to type II interferon	PMID:21873635	P
involved_in	GO:0071347	cellular response to interleukin-1	PMID:21873635	P
involved_in	GO:0071356	cellular response to tumor necrosis factor	PMID:21873635	P
involved_in	GO:0030593	neutrophil chemotaxis	PMID:21873635	P
involved_in	GO:0002548	monocyte chemotaxis	PMID:21873635	P
involved_in	GO:0006954	inflammatory response	PMID:21873635	P
involved_in	GO:0007186	G protein-coupled receptor signaling pathway	PMID:21873635	P
enables	GO:0048020	CCR chemokine receptor binding	PMID:21873635	F
enables	GO:0008009	chemokine activity	GO_REF:0000002	F
located_in	GO:0005576	extracellular region	GO_REF:0000002	C
involved_in	GO:0006955	immune response	GO_REF:0000002	P
located_in	GO:0005615	extracellular space	GO_REF:0000043	C
located_in	GO:0005576	extracellular region	GO_REF:0000043	C
enables	GO:0005125	cytokine activity	GO_REF:0000043	F
involved_in	GO:0006935	chemotaxis	GO_REF:0000043	P
involved_in	GO:0006954	inflammatory response	GO_REF:0000043	P
enables	GO:0008009	chemokine activity	PMID:9743322	F
enables	GO:0005102	signaling receptor binding	PMID:8702936	F
enables	GO:0005102	signaling receptor binding	PMID:9743322	F
involved_in	GO:0006935	chemotaxis	PMID:9743322	P
involved_in	GO:0007267	cell-cell signaling	PMID:8702936	P
located_in	GO:0005576	extracellular region	Reactome:R-HSA-443986	C
enables	GO:0005515	protein binding	PMID:28381538	F
enables	GO:0005515	protein binding	PMID:28381538	F
enables	GO:0005515	protein binding	PMID:28381538	F
enables	GO:0005515	protein binding	PMID:28381538	F
enables	GO:0005515	protein binding	PMID:28381538	F
enables	GO:0005515	protein binding	PMID:28381538	F
enables	GO:0005515	protein binding	PMID:28381538	F
enables	GO:0005515	protein binding	PMID:28381538	F
enables	GO:0005515	protein binding	PMID:28381538	F
involved_in	GO:0045662	negative regulation of myoblast differentiation	GO_REF:0000107	P
enables	GO:0031729	CCR4 chemokine receptor binding	GO_REF:0000107	F
located_in	GO:0005576	extracellular region	GO_REF:0000044	C
involved_in	GO:0061844	antimicrobial humoral immune response mediated by antimicrobial peptide	PMID:12949249	P
involved_in	GO:0031640	killing of cells of another organism	PMID:12949249	P
involved_in	GO:0031640	killing of cells of another organism	PMID:12949249	P

TIMP-3

Qualifier	GO Term	GO Name	Reference	GO Aspect
enables	GO:0002020	protease binding	PMID:21873635	F
enables	GO:0008191	metalloendopeptidase inhibitor activity	PMID:21873635	F
involved_in	GO:0051045	negative regulation of membrane protein	PMID:21873635	P

		ectodomain proteolysis		
is_active_in	GO:0005615	extracellular space	PMID:21873635	C
involved_in	GO:0009725	response to hormone	PMID:21873635	P
involved_in	GO:0010033	response to organic substance	PMID:21873635	P
involved_in	GO:0010951	negative regulation of endopeptidase activity	PMID:21873635	P
is_active_in	GO:0031012	extracellular matrix	PMID:21873635	C
involved_in	GO:0034097	response to cytokine	PMID:21873635	P
enables	GO:0008191	metalloendopeptidase inhibitor activity	GO_REF:0000002	F
enables	GO:0008191	metalloendopeptidase inhibitor activity	GO_REF:0000043	F
involved_in	GO:0050896	response to stimulus	GO_REF:0000043	P
enables	GO:0030414	peptidase inhibitor activity	GO_REF:0000043	F
involved_in	GO:0007601	visual perception	GO_REF:0000043	P
involved_in	GO:0010466	negative regulation of peptidase activity	GO_REF:0000043	P
located_in	GO:0005576	extracellular region	GO_REF:0000043	C
enables	GO:0004857	enzyme inhibitor activity	GO_REF:0000043	F
enables	GO:0046872	metal ion binding	GO_REF:0000043	F
located_in	GO:0005576	extracellular region	Reactome:R-HSA-481009	C
located_in	GO:0031089	platelet dense granule lumen	Reactome:R-HSA-481009	C
enables	GO:0005515	protein binding	PMID:18344519	F
involved_in	GO:0071310	cellular response to organic substance	GO_REF:0000107	P
located_in	GO:0005604	basement membrane	GO_REF:0000107	C
involved_in	GO:1904684	negative regulation of metalloendopeptidase activity	GO_REF:0000107	P
enables	GO:0008191	metalloendopeptidase inhibitor activity	PMID:18638486	F
involved_in	GO:1903984	positive regulation of TRAIL-activated apoptotic signaling pathway	PMID:19962668	P
part_of	GO:0062023	collagen-containing extracellular matrix	PMID:25037231	C
part_of	GO:0062023	collagen-containing extracellular matrix	PMID:28675934	C
part_of	GO:0062023	collagen-containing extracellular matrix	PMID:25037231	C
located_in	GO:0062023	collagen-containing extracellular matrix	PMID:27559042	C
located_in	GO:0062023	collagen-containing extracellular matrix	PMID:27068509	C
colocalizes_with	GO:0062023	collagen-containing extracellular matrix	PMID:20551380	C
involved_in	GO:0051045	negative regulation of membrane protein ectodomain proteolysis	PMID:18383040	P
involved_in	GO:0070373	negative regulation of ERK1 and ERK2 cascade	PMID:19962668	P
involved_in	GO:1904684	negative regulation of metalloendopeptidase activity	GO_REF:0000024	P
enables	GO:0005515	protein binding	PMID:18638486	F
located_in	GO:0005634	nucleus	PMID:21630459	C

NID1

Qualifier	GO Term	GO Name	Reference	GO Aspect
is_active_in	GO:0005886	plasma membrane	PMID:21873635	C
enables	GO:0005509	calcium ion binding	GO_REF:0000002	F
involved_in	GO:0007160	cell-matrix adhesion	GO_REF:0000002	P
located_in	GO:0005576	extracellular region	GO_REF:0000043	C

located_in	GO:0005604	basement membrane	GO_REF:0000043	C
involved_in	GO:0007155	cell adhesion	GO_REF:0000043	P
located_in	GO:0005576	extracellular region	Reactome:R-HSA-2426450	C
located_in	GO:0005576	extracellular region	Reactome:R-HSA-3791319	C
enables	GO:0005518	collagen binding	GO_REF:0000107	F
involved_in	GO:0010811	positive regulation of cell-substrate adhesion	GO_REF:0000107	P
located_in	GO:0071944	cell periphery	GO_REF:0000107	C
located_in	GO:0031012	extracellular matrix	GO_REF:0000107	C
located_in	GO:0005604	basement membrane	GO_REF:0000107	C
involved_in	GO:0030198	extracellular matrix organization	GO_REF:0000107	P
involved_in	GO:0032836	glomerular basement membrane development	GO_REF:0000107	P
involved_in	GO:0007160	cell-matrix adhesion	GO_REF:0000107	P
enables	GO:0043237	laminin-1 binding	GO_REF:0000107	F
enables	GO:0050840	extracellular matrix binding	GO_REF:0000107	F
located_in	GO:0005604	basement membrane	PMID:22952693	C
enables	GO:0005518	collagen binding	PMID:22952693	F
enables	GO:0043236	laminin binding	PMID:22952693	F
involved_in	GO:0071711	basement membrane organization	PMID:22952693	P
enables	GO:0005201	extracellular matrix structural constituent	PMID:25037231	F
enables	GO:0005201	extracellular matrix structural constituent	PMID:25037231	F
enables	GO:0005201	extracellular matrix structural constituent	PMID:28675934	F
enables	GO:0005201	extracellular matrix structural constituent	PMID:20551380	F
enables	GO:0005201	extracellular matrix structural constituent	PMID:27559042	F
enables	GO:0005201	extracellular matrix structural constituent	PMID:27068509	F
part_of	GO:0062023	collagen-containing extracellular matrix	PMID:25037231	C
part_of	GO:0062023	collagen-containing extracellular matrix	PMID:25037231	C
part_of	GO:0062023	collagen-containing extracellular matrix	PMID:28675934	C
located_in	GO:0062023	collagen-containing extracellular matrix	PMID:27559042	C
located_in	GO:0062023	collagen-containing extracellular matrix	PMID:27068509	C
colocalizes_with	GO:0062023	collagen-containing extracellular matrix	PMID:20551380	C
colocalizes_with	GO:0062023	collagen-containing extracellular matrix	PMID:22261194	C
enables	GO:0005201	extracellular matrix structural constituent	GO_REF:0000024	F
enables	GO:0043394	proteoglycan binding	PMID:22952693	F
located_in	GO:0005604	basement membrane	GO_REF:0000044	C
involved_in	GO:0110011	regulation of basement membrane organization	PMID:2119632	P
involved_in	GO:0045785	positive regulation of cell adhesion	PMID:3794389	P
involved_in	GO:0051149	positive regulation of muscle cell differentiation	PMID:26555376	P
located_in	GO:0098637	protein complex involved in cell-matrix adhesion	PMID:3109910	C
involved_in	GO:2001046	positive regulation of integrin-mediated signaling pathway	PMID:23948589	P
located_in	GO:0005604	basement membrane	PMID:1678389	C
located_in	GO:0070062	extracellular exosome	PMID:23533145	C
located_in	GO:0070062	extracellular exosome	PMID:19056867	C
located_in	GO:0062023	collagen-containing extracellular matrix	PMID:23658023	C

located_in	GO:0110165	cellular anatomical entity	GO_REF:0000117	C

NID2

Qualifier	GO Term	GO Name	Reference	GO Aspect
is_active_in	GO:0005615	extracellular space	PMID:21873635	C
is_active_in	GO:0005604	basement membrane	PMID:21873635	C
involved_in	GO:0007160	cell-matrix adhesion	PMID:21873635	P
enables	GO:0005509	calcium ion binding	GO_REF:0000002	F
involved_in	GO:0007160	cell-matrix adhesion	GO_REF:0000002	P
located_in	GO:0005576	extracellular region	GO_REF:0000043	C
located_in	GO:0005604	basement membrane	GO_REF:0000043	C
involved_in	GO:0007155	cell adhesion	GO_REF:0000043	P
enables	GO:0005518	collagen binding	PMID:9733643	F
located_in	GO:0005604	basement membrane	PMID:9733643	C
involved_in	GO:0007155	cell adhesion	PMID:9733643	P
located_in	GO:0005576	extracellular region	Reactome:R-HSA-2426450	C
located_in	GO:0031012	extracellular matrix	GO_REF:0000107	C
located_in	GO:0005604	basement membrane	GO_REF:0000107	C
involved_in	GO:0007160	cell-matrix adhesion	GO_REF:0000107	P
located_in	GO:0005604	basement membrane	PMID:22952693	C
involved_in	GO:0071711	basement membrane organization	PMID:22952693	P
enables	GO:0005201	extracellular matrix structural constituent	PMID:28675934	F
enables	GO:0005201	extracellular matrix structural constituent	PMID:20551380	F
enables	GO:0005201	extracellular matrix structural constituent	PMID:27559042	F
enables	GO:0005201	extracellular matrix structural constituent	PMID:27068509	F
enables	GO:0005201	extracellular matrix structural constituent	PMID:28327460	F
part_of	GO:0062023	collagen-containing extracellular matrix	PMID:28675934	C
located_in	GO:0062023	collagen-containing extracellular matrix	PMID:28327460	C
located_in	GO:0062023	collagen-containing extracellular matrix	PMID:27559042	C
located_in	GO:0062023	collagen-containing extracellular matrix	PMID:27068509	C
colocalizes_with	GO:0062023	collagen-containing extracellular matrix	PMID:20551380	C
enables	GO:0005201	extracellular matrix structural constituent	GO_REF:0000024	F
located_in	GO:0005604	basement membrane	GO_REF:0000044	C
located_in	GO:0005886	plasma membrane	GO_REF:0000052	C
located_in	GO:0070062	extracellular exosome	PMID:23533145	C
located_in	GO:0062023	collagen-containing extracellular matrix	PMID:23658023	C
enables	GO:0005515	protein binding	PMID:11956183	F
located_in	GO:0110165	cellular anatomical entity	GO_REF:0000117	C

Table S8. ROC curve data of ALS, S-ALS and F-ALS versus controls (H-CTR and NoH-CTR).

ALS versus controls

Protein Name	AUC	P value	Z score	Sensitivity	Specificity	Optimal cut-off point
TARC	0.68	0.0034	2.93	0.56	0.72	57.22
TIMP3	0.60	0.14194	1.47	-	-	-
NID1	0.52	0.78816	0.27	-	-	-
NID2	0.58	0.22565	1.27	-	-	-

S-ALS versus controls

Protein Name	AUC	P value	Z score	Sensitivity	Specificity	Optimal cut-off point
TARC	0.67	0.00915	2.60	0.562	0.722	57.22
TIMP3	0.67	0.00827	2.64	0.656	0.694	49.4
NID1	0.54	0.55898	0.58	-	-	-
NID2	0.51	0.89388	0.13	-	-	-

F-ALS versus controls

Protein Name	AUC	P value	Z score	Sensitivity	Specificity	Optimal cut-off point
TARC	0.72	0.00856	2.63	1	0.375	31.49
TIMP3	0.65	0.1389	1.48.			
NID1	0.53	0.79092	0.26.			
NID2	0.81	0.00002	4.32.	0.583	0.938	22.74

Table S9. Stepwise logistic regression analysis of ALS, S-ALS and F-ALS versus controls (H-CTR and NoH-CTR). Abbreviations: VIF-Variance Inflation Factor

ALS versus controls

Protein Name	P value	VIF
TARC	0.00836	1.360
NID1	0.03857	1.360

S-ALS versus controls

Protein Name	P value	VIF
TARC	0.00240	1.428
NID1	0.05980	1.428

F-ALS versus controls

Protein Name	P value	VIF
TARC	0.07890	1.113
TIMP3	0.02540	1.087
NID2	0.02120	1.026
